# Fibrous dysplasia and aneurysmal bone cyst of the skull base presenting with blindness: a report of a rare locally aggressive example

**DOI:** 10.1186/1758-3284-3-15

**Published:** 2011-03-11

**Authors:** Abdullah Sulieman Terkawi, Khalid H Al-Qahtani, Eman Baksh, Lahbib Soualmi, Asim El-Bagir Mohamed, Abdulrahman J Sabbagh

**Affiliations:** 1 Department of Otolaryngology - Head and Neck Surgery, King Fahad Medical City, Riyadh - Saudi Arabia; 2 Department of Radiology, King Fahad Medical City, Riyadh - Saudi Arabia; 3 Department of Neurosurgery, King Fahad Medical City, Riyadh - Saudi Arabia; 4 Department of Pathology, King Fahad Medical City, Riyadh - Saudi Arabia

## Abstract

Fibrous dysplasia (FD) and aneurysmal bone cyst (ABC) are uncommon benign intraosseous lesions. Simultaneous occurrence of both lesions is extremely rare. We present an example of concomitant FD and ABC in a 7 year-old with left eye blindness and discharge of one month duration. Physical examination revealed a proptotic left eye and bulging of the hard palate. CT and MRI are consistent with FD and ABC that involved the sphenoid and ethmoidal bones bilaterally. Incomplete combined endonasalcranial resection was performed. The patient presented five months postoperatively with a large recurrence and subsequent follow up was lost. Concomitant FD with ABC may occur in paranasal sinuses and may develop rapidly and exhibit locally aggressive behavior.

## Introduction

Fibrous dysplasia (FD) consists of rare and benign osseous lesions of unknown etiology. They represent 2.5% of all bone tumors and 7% of benign bone tumors [1], in young, predominantly male patients [2,3]. The salient pathological feature of FD consists of the replacement of medullary bone by histologically benign fibro-osseous tissue [2]. FD may present as monostotic (70%), polystotic (30%), or as the main feature of McCune-Albright syndrome [4]. Craniofacial bones can be affected by monostotic (25%-30%) and polystatic (in 50%) lesions [5]. The most affected craniofacial bones are maxilla, mandible, frontal, sphenoid and temporal bones [6].

Aneurysmal bone cyst (ABC) is rare, benign vascular lesion, and considered secondary to certain pathological bone lesions [7]. ABC represents approximately 1.4% of all bone tumors, and only 3% among those are located in the cranium [8]. These lesions occur most commonly in patients under 20 years old of both genders [8]. Radiographs reveal an eccentric, lytic lesion typically with an expanded, remodeled "blown-out" or "ballooned" bony contour of the affected bone, with a delicate trabeculated appearance frequently. Fluid-filled spaces are common and may be seen on CT scans and MR images [7].

Concomitant FD and ABC are extremely rare with only 13 cases reported previously [9-12]. The clinical presentation of both FD and ABC depends on their location and the extent of involvement. The majority of reported cases are predominant in male children and young adults that are presented with symptomatic or asymptomatic mass. The development of ABC in FB may hastens the course of presentation [12]. In general, a complete excision of these lesions is recommended, and reports of aggressive behavior are occasionally found.

We report a rare example of concomitant FD and ABC of the skull base that presented with blindness and rapid local recurrence in a 7 year-old female patient. Also, we are presenting the multidisciplinary team approach of treating such cases.

## Case report

A 7 year-old female, is presented at a local hospital with left eye blindness and discharge of one-month duration.

**Figure 1:**
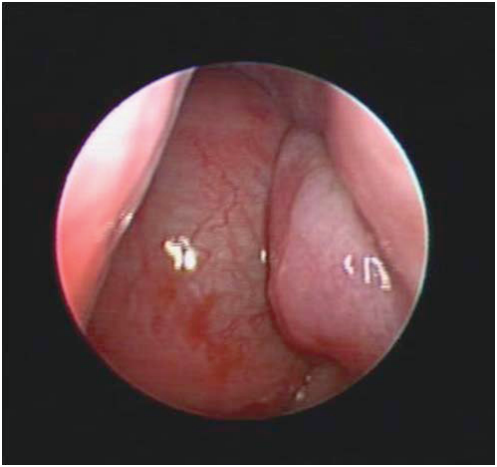
**Endoscopic nasal view of left nostril, revealed huge nasal mass that occupied the whole cavity and reach to the level of inferior turbinate**.

Physical examination showed a slightly proptotic left eye with no light perception, absence of direct pupil reflex and optic nerve atrophy in the left eye. In addition to, a slight hypertelorism. Otolaryngologic examination showed rhinorhea, bulging hard palate, and a left nasal mass (Figure 1).

**Figure 2:**
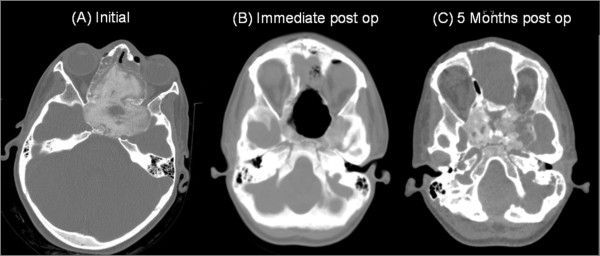
**Series CT scans of the tumor.** (A) preoperative CT revealing ground glass appearance is consistent with fibrous dysplasia affecting of the sphenoid bone, ethmoidial bones, LT maxillary sinus, involving the optic foramen and harboring cystic expansible component. (B) Immediate post operative CT scan. (C) 5 months post operative follow up CT scan.

**Figure 3:**
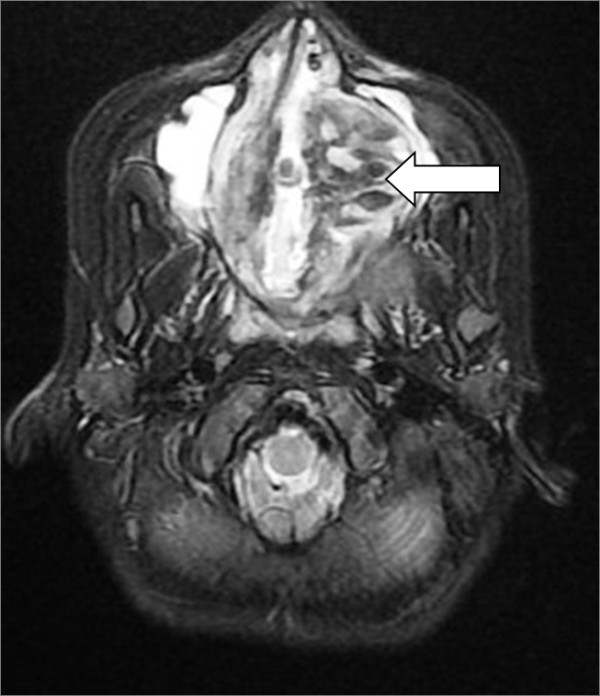
**Preoperative Axial T2 of the skull base revealed multiple fluid-fluid levels with hemorrhagic areas (white arrow) are consistent with associated aneurysmal bone cyst**.

Preoperative CT findings (Figure 2, A) were consistent with fibrous dysplasia involving the sphenoid, ethmoidal sinuses, and posterior aspect of the nasal cavity as well as, the left maxillary sinus and the left optic foramen. Large expansible cystic spaces were also noticed in the lesion on preoperative MRI (Figure 3). Endoscopic biopsies revealed fibrous dysplasia with vascular component that were associated with significant and difficult to control bleeding.

### Surgical Excision

A combined endonasal-cranial approach to resect this lesion that entailed an extradural bi-coronal craniotomy (pericranium saving technique for closure) and medial bilateral orbitotomy was performed (Figure 2, B). The preoperative assessment of the tumor (using the neuronavigation system) revealed a volume of 88.67 cc, approximately 95% (84.17 cc) of the tumor was removed. 4.5 cc was left over the right optic nerve to preserve the vision on the unaffected eye. The intraoperative blood loss was estimated about 1700 cc. And the patient recovered without any appreciable complications, along with improvement of her proptosis. She was discharged with clinical and imaging follow up.

### Histopathology

**Figure 4:**
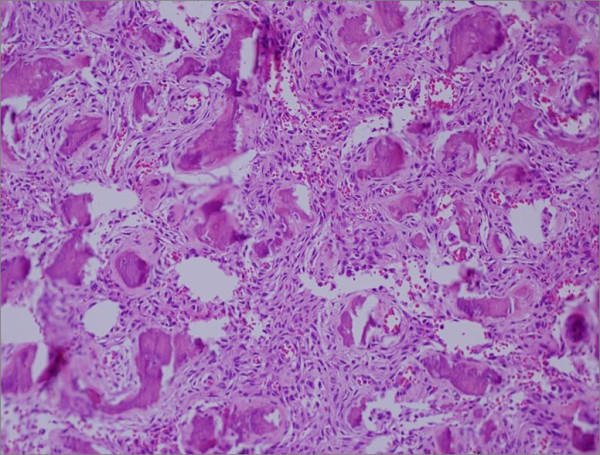
**Fibrous dysplasia**. Photomicrograph reveling spindle cells proliferation with islands of mature bone structure (with Chinese like characters). No mitosis or cellular features of malignancy are presented.

**Figure 5:**
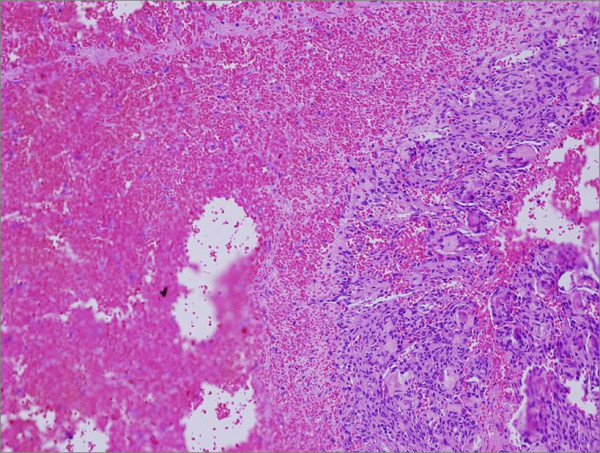
**Aneurysmal bone cyst**. Photomicrograph shows spindle cell proliferation with giant cell lining bloody cystic structure.

Histological evaluation revealed fibroblastic proliferation comprised of benign fibrous spindle areas with mature irregular bone formation (Figure 4). Cystic formation and hemorrhagic spaces lined by osteoclast-like multinucleated giant were also noted (Figure 5). The findings were consisted of fibrous dysplasia with aneurismal bone cyst-like features.

### Follow-up

Five months after surgery, a recurrent large tumor was noticed, estimated to be 30.5 cc (Figure 2, C). The patient's parents declined any further treatment, and the patient was lost to follow up.

## Discussion

We present a rare case of FD with ABC in the skull base of a female child with sudden blindness. The radiological and pathological findings were consistent with the diagnosis in this case. In contrast to previous reports, however, the present case progressed rapidly and led to bone destruction and extension to adjacent vital structures. Saito et al [12] presented a similar case involving the nasal cavity and the sphenoid sinus with symptoms that preceded the diagnosis by one year. In their case, complete excision was achieved with no recurrence after 3.5 years of follow up. Of the [11] concomitant FD and ABC that have been reported (Table 1[8-18]), [9,13,14,16-18], three occurred in the sinonasal cavity [8,12,13,15].

**Table 1:** Clinical, radiological findings, and treatment of reported combined fibrous dysplasia and aneurysmal bone cyst of the skull

Reference	Age (years) Gender	Site	Clinical presentation	Radiological findings	Treatment/Surgical approach	Follow-up
**Składzień et al., 2008 **[8]	16/M	Rt Maxillary, orbital	Epistaxis and chronic rhinosinusitis	Large cystic lesion	En-bloc excision	9 mo disease free
**Rappaport, 1989 **[9]	25/M	Lt occipital	Painful mass	CT: pagetoid changes and hypodense lesion	Surgical excision	No f/u
**Wojno and McCathy, 1994 **[10]	14/F	Rt temporal	Painless mass	CT: nonhomogeneous cystic lesion	Surgical excision	2 yr disease free
**Wojno and McCathy, 1994 **[10]	40/M	Lt frontal	Expanding mass Mass appear after head trauma	CT: diffuse thickening of the calvarium, Lt frontal cyst	Surgical excision	No f/u
**Haddad et al., 1998 **[11]	6.5/M	Rt temporal	Rt painless temporal mass	CT: nonhomogeneous cystic mass.	Frontotemporal excision	4 yr disease free
**Saito et al. 1998 **[12]	11/M	nasal cavity, sphenoid bone, and skull base	Nasal obstruction and headache for 1 yr	CT and MRI, irregular multilobulated tumor	Craniofacial excision	3.5 yr disease free
**Branch et al., 1986 **[13]	9/F	Lt parietal & fronto-temporal	Painful Lt and frontal parietal mass for 1 mo	CT: large cystic bone lesion in the parietal area	Surgical excision	No f/u
**Branch et al., 1986 **[13]	19/M	Rt parietal	Painless mass k/c of FD for 15 yr	CT: cystic expansion of the skull	Surgical excision	No f/u
**Itshayek et al., 2002 **[14]	19/M	Lt occipital bone and clivus	Painless mass of the occipital area	CT: occipital cyst lesion	Selective embolization. Followed by surgical resection.	1 yr disease free
**Pasquini et al., 2002 **[15]	5/M	Rt maxillary sinus	Progressive and persistent Rt side epiphora and rhinosinusitis for 2 yr.	CT: Cyst-like lesion.	Transnasal endoscopic surgery	No f/u
**Lin et al., 2004 **[16]	18/M	Lt frontal bone	Progressive enlargement of the mass with severe headache	CT: several expansile cystic spaces	Surgical resection	No f/u
**Iseri et al., 2005 **[17]	35/F	Lt occipital bone	Progressive severe headache	FD of clivus, temporal, and occipital bones.	Unresectable	
**Mattei et al., 2005 **[18]	19/M	Occipital bone	As SAH; severe headache and nuchal rigidity.	Hemmorage and cyst	Partial surgical resection	No f/u
**Our case**	7/F	Sphenoidal and ethmoidal bones	Lt eye loss of vision Lt nasal obstruction	Cystic expansible lesion	Endonasal - cranial resection	Recur after 5 mo

FD; fibrous dysplasia. AG; angiogram. LPMA; Lt posterior meningeal artery. SAH; subarachnoid hemorrhage. ABC; aneurysmal bone cyst. f/u; follow up. k/c; known case.

PNS; paranasal sinuses.

The presentation of these lesions, as in our case, depends on the location, rate of growth and site of involvement. Symptoms may include painless mass, pressure symptoms, nasal obstruction, headache and loss of vision. Acute hemorrhage into the cyst(s) may causes pain, rapid enlargement and/or rupture and subarachnoid hemorrhage [18]. All reported cases were treated surgically with generally good outcomes.

ABC in distal tibia has been treated by high-energy, low-dose radiation on one patient and was successful, with no recurrence during the five years follow up [19]. However, radiation for ABC of skull base or concomitant ABC with FD residual tumors has not been reported. The aggressive nature of the present case and others suggests that adjuvant therapy may be required in some cases.

## Conclusion

Our case represents a rapid developing fibrous dysplasia with aneurismal bone cyst at the skull base leading to unilateral blindness. Awareness of such cases may lead to early detection and diagnosis for effective therapy.

A multidisciplinary team is needed for both diagnosis (clinical, radiological, and histopathological) and management (otolaryngology, neurosurgery, and neuronavigation) in this kind of diseases.

## Competing interests

The authors declare that they have no competing interests.

## Authors' contributions

AST: writer, alignment and drafted the manuscript. KHQ: surgen, writer. EB: radiological diagnosis, writer. LS: neuronavigation, writer. ABM: pathological diagnosis, writer. AJS: surgen, writer. All authors read and approved the final manuscript.

## Consent

Written informed consent was obtained from the patient for publication of this case report and accompanying images. A copy of the written consent is available for review by the Editor-in-Chief of this journal.
